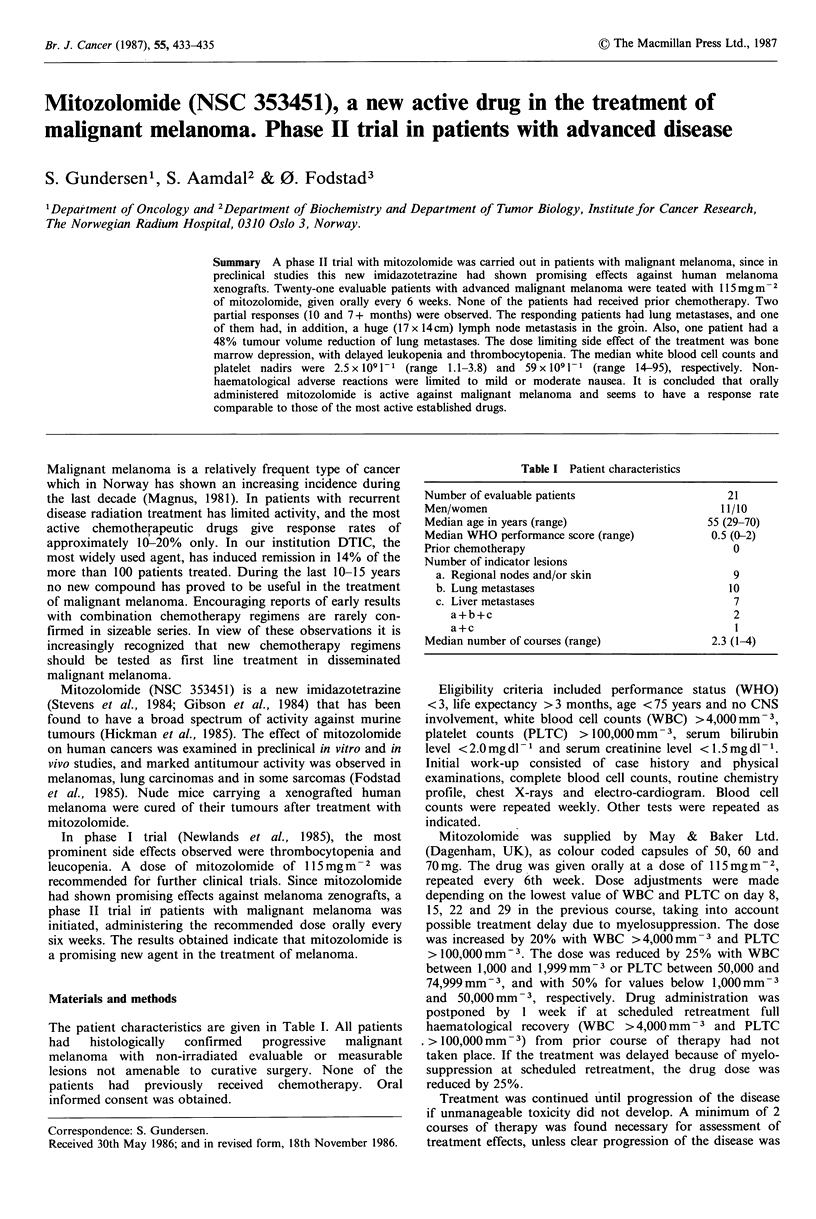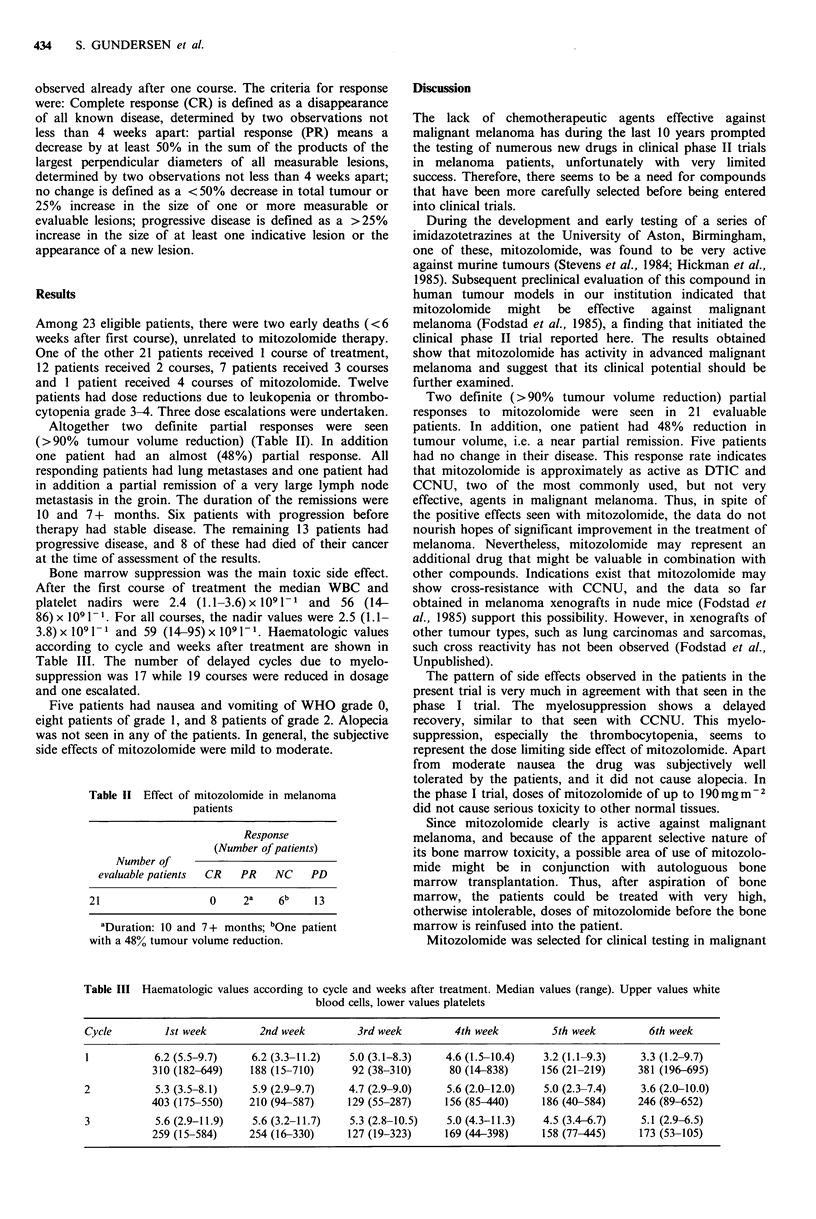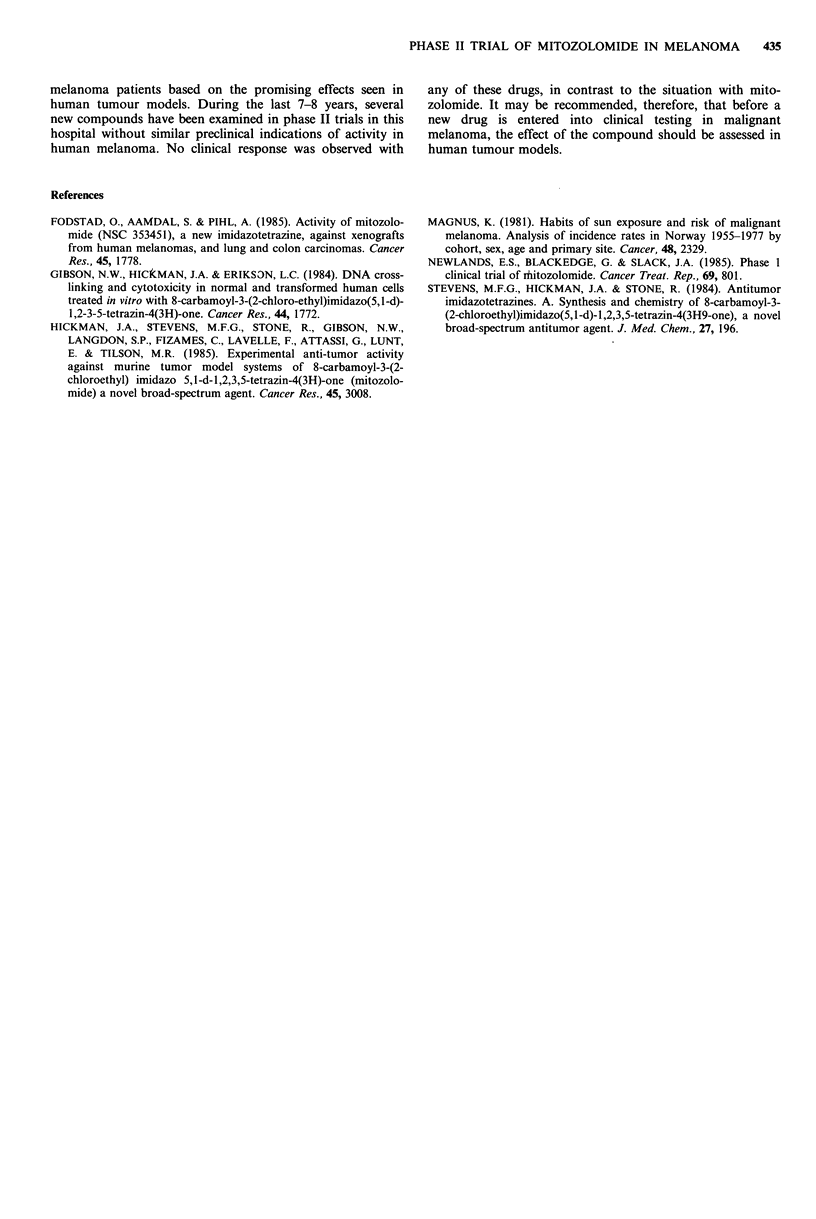# Mitozolomide (NSC 353451), a new active drug in the treatment of malignant melanoma. Phase II trial in patients with advanced disease.

**DOI:** 10.1038/bjc.1987.85

**Published:** 1987-04

**Authors:** S. Gundersen, S. Aamdal, O. Fodstad

## Abstract

A phase II trial with mitozolomide was carried out in patients with malignant melanoma, since in preclinical studies this new imidazotetrazine had shown promising effects against human melanoma xenografts. Twenty-one evaluable patients with advanced malignant melanoma were treated with 115 mg m-2 of mitozolomide, given orally every 6 weeks. None of the patients had received prior chemotherapy. Two partial responses (10 and 7+ months) were observed. The responding patients had lung metastases, and one of them had, in addition, a huge (17 X 14 cm) lymph node metastasis in the groin. Also, one patient had a 48% tumour volume reduction of lung metastases. The dose limiting side effect of the treatment was bone marrow depression, with delayed leukopenia and thrombocytopenia. The median white blood cell counts and platelet nadirs were 2.5 X 10(9) 1(-1) (range 1.1-3.8) and 59 X 10(9) 1(-1) (range 14-95), respectively. Non-haematological adverse reactions were limited to mild or moderate nausea. It is concluded that orally administered mitozolomide is active against malignant melanoma and seems to have a response rate comparable to those of the most active established drugs.


*Br. J. Cancer* (1987), **55**, 433–435 © The Macmillan Press Ltd., 1987

### Mitozolomide (NSC 353451), a new active drug in the treatment of malignant melanoma. Phase II trial in patients with advanced disease

S. Gundersen[1], S. Aamdal[2] & Ø. Fodstad[3]

[1]*Department of Oncology and* [2]*Department of Biochemistry and Department of Tumor Biology, Institute for Cancer Research, The Norwegian Radium Hospital, 0310 Oslo 3, Norway.*

**Summary** A phase II trial with mitozolomide was carried out in patients with malignant melanoma, since in preclinical studies this new imidazotetrazine had shown promising effects against human melanoma xenografts. Twenty-one evaluable patients with advanced malignant melanoma were teated with $115\,mg\,m^{-2}$ of mitozolomide, given orally every 6 weeks. None of the patients had received prior chemotherapy. Two partial responses (10 and 7+ months) were observed. The responding patients had lung metastases, and one of them had, in addition, a huge (17×14cm) lymph node metastasis in the groin. Also, one patient had a 48% tumour volume reduction of lung metastases. The dose limiting side effect of the treatment was bone marrow depression, with delayed leukopenia and thrombocytopenia. The median white blood cell counts and platelet nadirs were $2.5\times10^9\,l^{-1}$ (range 1.1–3.8) and $59\times10^9\,l^{-1}$ (range 14–95), respectively. Non-haematological adverse reactions were limited to mild or moderate nausea. It is concluded that orally administered mitozolomide is active against malignant melanoma and seems to have a response rate comparable to those of the most active established drugs.

Malignant melanoma is a relatively frequent type of cancer which in Norway has shown an increasing incidence during the last decade (Magnus, 1981). In patients with recurrent disease radiation treatment has limited activity, and the most active chemotherapeutic drugs give response rates of approximately 10–20% only. In our institution DTIC, the most widely used agent, has induced remission in 14% of the more than 100 patients treated. During the last 10–15 years no new compound has proved to be useful in the treatment of malignant melanoma. Encouraging reports of early results with combination chemotherapy regimens are rarely confirmed in sizeable series. In view of these observations it is increasingly recognized that new chemotherapy regimens should be tested as first line treatment in disseminated malignant melanoma.

Mitozolomide (NSC 353451) is a new imidazotetrazine (Stevens *et al.*, 1984; Gibson *et al.*, 1984) that has been found to have a broad spectrum of activity against murine tumours (Hickman *et al.*, 1985). The effect of mitozolomide on human cancers was examined in preclinical *in vitro* and *in vivo* studies, and marked antitumour activity was observed in melanomas, lung carcinomas and in some sarcomas (Fodstad *et al.*, 1985). Nude mice carrying a xenografted human melanoma were cured of their tumours after treatment with mitozolomide.

In phase I trial (Newlands *et al.*, 1985), the most prominent side effects observed were thrombocytopenia and leucopenia. A dose of mitozolomide of $115\,mg\,m^{-2}$ was recommended for further clinical trials. Since mitozolomide had shown promising effects against melanoma zenografts, a phase II trial in patients with malignant melanoma was initiated, administering the recommended dose orally every six weeks. The results obtained indicate that mitozolomide is a promising new agent in the treatment of melanoma.

### Materials and methods

The patient characteristics are given in Table I. All patients had histologically confirmed progressive malignant melanoma with non-irradiated evaluable or measurable lesions not amenable to curative surgery. None of the patients had previously received chemotherapy. Oral informed consent was obtained.

Correspondence: S. Gundersen.
Received 30th May 1986; and in revised form, 18th November 1986.

**Table I** Patient characteristics

| | |
|---|---|
| Number of evaluable patients | 21 |
| Men/women | 11/10 |
| Median age in years (range) | 55 (29–70) |
| Median WHO performance score (range) | 0.5 (0–2) |
| Prior chemotherapy | 0 |
| Number of indicator lesions | |
| a. Regional nodes and/or skin | 9 |
| b. Lung metastases | 10 |
| c. Liver metastases | 7 |
| a+b+c | 2 |
| a+c | 1 |
| Median number of courses (range) | 2.3 (1–4) |

Eligibility criteria included performance status (WHO) <3, life expectancy >3 months, age <75 years and no CNS involvement, white blood cell counts (WBC) $>4{,}000\,mm^{-3}$, platelet counts (PLTC) $>100{,}000\,mm^{-3}$, serum bilirubin level $<2.0\,mg\,dl^{-1}$ and serum creatinine level $<1.5\,mg\,dl^{-1}$. Initial work-up consisted of case history and physical examinations, complete blood cell counts, routine chemistry profile, chest X-rays and electro-cardiogram. Blood cell counts were repeated weekly. Other tests were repeated as indicated.

Mitozolomide was supplied by May & Baker Ltd. (Dagenham, UK), as colour coded capsules of 50, 60 and 70mg. The drug was given orally at a dose of $115\,mg\,m^{-2}$, repeated every 6th week. Dose adjustments were made depending on the lowest value of WBC and PLTC on day 8, 15, 22 and 29 in the previous course, taking into account possible treatment delay due to myelosuppression. The dose was increased by 20% with WBC $>4{,}000\,mm^{-3}$ and PLTC $>100{,}000\,mm^{-3}$. The dose was reduced by 25% with WBC between 1,000 and $1{,}999\,mm^{-3}$ or PLTC between 50,000 and $74{,}999\,mm^{-3}$, and with 50% for values below $1{,}000\,mm^{-3}$ and $50{,}000\,mm^{-3}$, respectively. Drug administration was postponed by 1 week if at scheduled retreatment full haematological recovery (WBC $>4{,}000\,mm^{-3}$ and PLTC $>100{,}000\,mm^{-3}$) from prior course of therapy had not taken place. If the treatment was delayed because of myelosuppression at scheduled retreatment, the drug dose was reduced by 25%.

Treatment was continued until progression of the disease if unmanageable toxicity did not develop. A minimum of 2 courses of therapy was found necessary for assessment of treatment effects, unless clear progression of the disease was observed already after one course. The criteria for response were: Complete response (CR) is defined as a disappearance of all known disease, determined by two observations not less than 4 weeks apart: partial response (PR) means a decrease by at least 50% in the sum of the products of the largest perpendicular diameters of all measurable lesions, determined by two observations not less than 4 weeks apart; no change is defined as a $<50\%$ decrease in total tumour or 25% increase in the size of one or more measurable or evaluable lesions; progressive disease is defined as a $>25\%$ increase in the size of at least one indicative lesion or the appearance of a new lesion.

### Results

Among 23 eligible patients, there were two early deaths ($<6$ weeks after first course), unrelated to mitozolomide therapy. One of the other 21 patients received 1 course of treatment, 12 patients received 2 courses, 7 patients received 3 courses and 1 patient received 4 courses of mitozolomide. Twelve patients had dose reductions due to leukopenia or thrombocytopenia grade 3–4. Three dose escalations were undertaken.

Altogether two definite partial responses were seen ($>90\%$ tumour volume reduction) (Table II). In addition one patient had an almost (48%) partial response. All responding patients had lung metastases and one patient had in addition a partial remission of a very large lymph node metastasis in the groin. The duration of the remissions were 10 and 7+ months. Six patients with progression before therapy had stable disease. The remaining 13 patients had progressive disease, and 8 of these had died of their cancer at the time of assessment of the results.

Bone marrow suppression was the main toxic side effect. After the first course of treatment the median WBC and platelet nadirs were 2.4 (1.1–3.6) $\times 10^9 l^{-1}$ and 56 (14–86) $\times 10^9 l^{-1}$. For all courses, the nadir values were 2.5 (1.1–3.8) $\times 10^9 l^{-1}$ and 59 (14–95) $\times 10^9 l^{-1}$. Haematologic values according to cycle and weeks after treatment are shown in Table III. The number of delayed cycles due to myelosuppression was 17 while 19 courses were reduced in dosage and one escalated.

Five patients had nausea and vomiting of WHO grade 0, eight patients of grade 1, and 8 patients of grade 2. Alopecia was not seen in any of the patients. In general, the subjective side effects of mitozolomide were mild to moderate.

**Table II** Effect of mitozolomide in melanoma patients

| Number of evaluable patients | Response (Number of patients) CR | PR | NC | PD |
|---|---|---|---|---|
| 21 | 0 | 2[a] | 6[b] | 13 |

[a]Duration: 10 and 7+ months; [b]One patient with a 48% tumour volume reduction.

### Discussion

The lack of chemotherapeutic agents effective against malignant melanoma has during the last 10 years prompted the testing of numerous new drugs in clinical phase II trials in melanoma patients, unfortunately with very limited success. Therefore, there seems to be a need for compounds that have been more carefully selected before being entered into clinical trials.

During the development and early testing of a series of imidazotetrazines at the University of Aston, Birmingham, one of these, mitozolomide, was found to be very active against murine tumours (Stevens et al., 1984; Hickman et al., 1985). Subsequent preclinical evaluation of this compound in human tumour models in our institution indicated that mitozolomide might be effective against malignant melanoma (Fodstad et al., 1985), a finding that initiated the clinical phase II trial reported here. The results obtained show that mitozolomide has activity in advanced malignant melanoma and suggest that its clinical potential should be further examined.

Two definite ($>90\%$ tumour volume reduction) partial responses to mitozolomide were seen in 21 evaluable patients. In addition, one patient had 48% reduction in tumour volume, i.e. a near partial remission. Five patients had no change in their disease. This response rate indicates that mitozolomide is approximately as active as DTIC and CCNU, two of the most commonly used, but not very effective, agents in malignant melanoma. Thus, in spite of the positive effects seen with mitozolomide, the data do not nourish hopes of significant improvement in the treatment of melanoma. Nevertheless, mitozolomide may represent an additional drug that might be valuable in combination with other compounds. Indications exist that mitozolomide may show cross-resistance with CCNU, and the data so far obtained in melanoma xenografts in nude mice (Fodstad et al., 1985) support this possibility. However, in xenografts of other tumour types, such as lung carcinomas and sarcomas, such cross reactivity has not been observed (Fodstad et al., Unpublished).

The pattern of side effects observed in the patients in the present trial is very much in agreement with that seen in the phase I trial. The myelosuppression shows a delayed recovery, similar to that seen with CCNU. This myelosuppression, especially the thrombocytopenia, seems to represent the dose limiting side effect of mitozolomide. Apart from moderate nausea the drug was subjectively well tolerated by the patients, and it did not cause alopecia. In the phase I trial, doses of mitozolomide of up to 190 mg m$^{-2}$ did not cause serious toxicity to other normal tissues.

Since mitozolomide clearly is active against malignant melanoma, and because of the apparent selective nature of its bone marrow toxicity, a possible area of use of mitozolomide might be in conjunction with autologuous bone marrow transplantation. Thus, after aspiration of bone marrow, the patients could be treated with very high, otherwise intolerable, doses of mitozolomide before the bone marrow is reinfused into the patient.

Mitozolomide was selected for clinical testing in malignant

**Table III** Haematologic values according to cycle and weeks after treatment. Median values (range). Upper values white blood cells, lower values platelets

| Cycle | 1st week | 2nd week | 3rd week | 4th week | 5th week | 6th week |
|---|---|---|---|---|---|---|
| 1 | 6.2 (5.5–9.7)<br>310 (182–649) | 6.2 (3.3–11.2)<br>188 (15–710) | 5.0 (3.1–8.3)<br>92 (38–310) | 4.6 (1.5–10.4)<br>80 (14–838) | 3.2 (1.1–9.3)<br>156 (21–219) | 3.3 (1.2–9.7)<br>381 (196–695) |
| 2 | 5.3 (3.5–8.1)<br>403 (175–550) | 5.9 (2.9–9.7)<br>210 (94–587) | 4.7 (2.9–9.0)<br>129 (55–287) | 5.6 (2.0–12.0)<br>156 (85–440) | 5.0 (2.3–7.4)<br>186 (40–584) | 3.6 (2.0–10.0)<br>246 (89–652) |
| 3 | 5.6 (2.9–11.9)<br>259 (15–584) | 5.6 (3.2–11.7)<br>254 (16–330) | 5.3 (2.8–10.5)<br>127 (19–323) | 5.0 (4.3–11.3)<br>169 (44–398) | 4.5 (3.4–6.7)<br>158 (77–445) | 5.1 (2.9–6.5)<br>173 (53–105) |

melanoma patients based on the promising effects seen in human tumour models. During the last 7–8 years, several new compounds have been examined in phase II trials in this hospital without similar preclinical indications of activity in human melanoma. No clinical response was observed with any of these drugs, in contrast to the situation with mitozolomide. It may be recommended, therefore, that before a new drug is entered into clinical testing in malignant melanoma, the effect of the compound should be assessed in human tumour models.

### References

FODSTAD, O., AAMDAL, S. & PIHL, A. (1985). Activity of mitozolomide (NSC 353451), a new imidazotetrazine, against xenografts from human melanomas, and lung and colon carcinomas. *Cancer Res.*, **45**, 1778.

GIBSON, N.W., HICKMAN, J.A. & ERIKSON, L.C. (1984). DNA cross-linking and cytotoxicity in normal and transformed human cells treated *in vitro* with 8-carbamoyl-3-(2-chloro-ethyl)imidazo(5,1-d)-1,2-3-5-tetrazin-4(3H)-one. *Cancer Res.*, **44**, 1772.

HICKMAN, J.A., STEVENS, M.F.G., STONE, R., GIBSON, N.W., LANGDON, S.P., FIZAMES, C., LAVELLE, F., ATTASSI, G., LUNT, E. & TILSON, M.R. (1985). Experimental anti-tumor activity against murine tumor model systems of 8-carbamoyl-3-(2-chloroethyl) imidazo 5,1-d-1,2,3,5-tetrazin-4(3H)-one (mitozolomide) a novel broad-spectrum agent. *Cancer Res.*, **45**, 3008.

MAGNUS, K. (1981). Habits of sun exposure and risk of malignant melanoma. Analysis of incidence rates in Norway 1955–1977 by cohort, sex, age and primary site. *Cancer*, **48**, 2329.

NEWLANDS, E.S., BLACKEDGE, G. & SLACK, J.A. (1985). Phase 1 clinical trial of mitozolomide. *Cancer Treat. Rep.*, **69**, 801.

STEVENS, M.F.G., HICKMAN, J.A. & STONE, R. (1984). Antitumor imidazotetrazines. A. Synthesis and chemistry of 8-carbamoyl-3-(2-chloroethyl)imidazo(5,1-d)-1,2,3,5-tetrazin-4(3H9-one), a novel broad-spectrum antitumor agent. *J. Med. Chem.*, **27**, 196.